# An updated checklist of Nepalese ants (Hymenoptera, Formicidae)

**DOI:** 10.3897/zookeys.1006.58808

**Published:** 2020-12-22

**Authors:** Indra Prasad Subedi, Prem Bahadur Budha, Himender Bharti, Leeanne Alonso

**Affiliations:** 1 Central Department of Zoology, Tribhuvan University, Kathmandu, Nepal Tribhuvan University Kathmandu Nepal; 2 Department of Zoology and Environmental Sciences, Punjabi University, Patiala, India Punjabi University Patiala India; 3 Global Wildlife Conservation, Austin, USA Global Wildlife Conservation Austin United States of America

**Keywords:** Endemic, Himalaya, *
Myrmica
*, Nepal, *
Strumigenys
*, type locality

## Abstract

The location of Nepal in the Central Himalaya promotes high habitat and species diversity. Ant diversity is likely high, but there have been few studies of the diversity and distribution of ants in Nepal. Here we present an updated checklist list of Nepalese ants that includes 128 named species in 48 genera and eight subfamilies. Among these species, 21 species have a type locality from Nepal, nine species are endemic to Nepal, and three are introduced species. We add six new ant records for Nepal, namely *Harpegnathos venator*, *Monomorium pharaonis*, *Nylanderia bourbonica*, *Odontoponera denticulata*, *Polyrhachis tyrannica* and *Pseudoneoponera bispinosa*. The checklist presents distribution records for Nepalese ant species and provides comparisons with the neighboring countries of China and India.

## Introduction

Ants (family Formicidae) are one of the most successful groups of organisms on the planet (Hölldobler and Wilson 1990) and together with termites, have been found to make up 30% of animal biomass in the Amazon rain forest (Fittkau and Klinge 1973). Ants are present in almost all terrestrial ecosystems, with the peak of their diversity found within the tropical regions (Guénard 2013). Estimate of global ant species richness exceeds 20,000 species (Hölldobler and Wilson 1990). Seventeen valid subfamilies, 337 genera, and over 13,837 species of ants have been described (Bolton 2020). Over 87% of all described ant species belong to four main subfamilies: Myrmicinae, Formicinae, Ponerinae, and Dolichoderinae (Guénard 2013). Global Ant Biodiversity Informatics (GABI) is the first comprehensive global database with ant species records in available publications and existing databases (Guénard et al. 2017).

Nepal is a Himalayan country located in the Oriental region at the junction of the Palaearctic and Palaeotropic regions. Nepal covers an area of 147,516 km^2^ and lies in the center of the Himalayas between latitudes 26°28.986'N to 29°50.4726'N and longitudes 80°19.9998'E to 88°5.6616'E. Its unique geographical and ecological diversity promote a high diversity of flora and fauna, including of ants. However, the study of ants in Nepal is still in its infancy. The diversity and distribution of ants in Nepal is little known and there are few published records of ants of Nepal.

Early compilations of the ants of the Indian region by Bingham (1903) and of Asia by Chapman and Capco (1951) did not include ants from Nepal. The first reports of Nepalese ants were *Aphaenogaster pachei* and *Myrmica pachei* by Forel (1906). The next record of Nepalese ants appeared in Menozzi (1939). Collingwood (1970) published the first list of 34 species of ants of Nepal from the collections of the 1954 British Museum expedition to east Nepal and from Professor H Janetshek’s 1961 expedition to the Khumbu Himal region. Summaries of the insects of Nepal by Thapa (2000, 2015) listed 44 species (24 genera) of ants and 52 species (44 named species; 29 genera) of ants, respectively. The number of Nepalese ant species reported in online ant databases ranges from 57 named species (AntWeb 2020) to 86 species/subspecies (AntWiki 2020) to 141 species (antmaps.org 2020) but some of the records need verification of their occurrences in Nepal.

Thus, the information available on the diversity and distribution of Nepalese ants is scattered and incomplete. As a first step towards filling information gaps, this paper presents a thorough review of publications on the ants of Nepal to provide an up-to-date checklist of Nepalese ants. We also add additional species records and distribution information from collections done by the first author. This paper further compares the occurrence of the Nepalese ants in the neighboring countries of China and India. This checklist provides a baseline for Nepalese ant diversity and will hopefully stimulate additional research on the myrmecofauna of Nepal.

## Materials and methods

The species list of the ants of Nepal has been synthesized from a literature review of articles, books and reports. Information was also extracted from the online ant databases, AntWeb (2020) and AntWiki (2020) and species distribution information from antmaps.org (Janicki et al. 2016; Guénard et al. 2017). Species described as morphospecies or unidentified species are not included in this checklist. The data presented here are based mainly on existing literature and are thus dependent on the quality of the identification made by the authors at the time the record was published. New records and additional distribution localities of Nepalese ants were provided from the first author’s collection, deposited at Central Department Zoology Museum of Tribhuvan University, Nepa, **CDZMTU**. Taxonomic changes made to some of the Nepalese ant taxa are also incorporated. The valid name, spelling and authority of ant species were updated from Bolton (2020).

The subfamilies, genera within a subfamily, and species within a genus are arranged alphabetically. For each genus, type species and type locality are also provided. The species list provides the distribution range within Nepal including district, specific locality, altitude, collectors’ names, and depositories of ant specimens based upon the availability of data. Separate lists of the ant species first described from Nepal, endemic species of Nepal, and introduced species are also presented. A comparison of the occurrence of Nepalese ants in the neighboring countries of China and India is also included, which could aid in assessing the patterns of diversity and composition of Nepalese ants. Previously reported species if not substantiated by specimens or literature records, are marked as dubious/unverified. They have been excluded from the main species list and presented in a separate list.

The acronyms of depositories mentioned in this checklist are listed below:

**ANIC**Australian National Insect Collection, Canberra, Australia;

**AMNH**American Museum of Natural History, New York City, USA;

**CASC**California Academy of Sciences, San Francisco, California;

**CDZMTU**Central Department Zoology Museum of Tribhuvan University, Nepal;

**ELMES** Collection of GW Elmes, UK;

**JTLC**John T Longino personal collection, University of Utah, Utah, USA;

**KGAC** Kiko Gómez Abal collection, Barcelona. Spain;

**MARTENS** University of Mainz, Germany (Private Collection of Martens);

**MCZC**Museum of Comparative Zoology, Harvard University, Cambridge, Massachusetts, USA;

**MHNG**Muséum d’Histoire Naturelle, Geneva, Switzerland;

**MSNG**Museo Civico di Storia Naturale “Giacomo Doria”, Genoa, Italy;

**NHMB**Naturhistorisches Museum, Basel, Switzerland;

**NHMUK**The Natural History Museum, London, United Kingdom;

**NHMW**The Natural History Museum Vienna, Austria;

**PSWC** Philip S Ward collection, California, USA;

**SCHULZ** Ted Schulz private collection, Washington DC, USA;

**SIZK**Schmalhausen Institute of Zoology of the Ukrainian National Academy of Sciences, Kiev;

**UCDC**Bohart Museum of Entomology, University of California, Davis;

**USNM**United States National Museum of Natural History, Washington DC.

## Results and discussion

The checklist of the ants of Nepal includes 128 valid species. These ant species represent ca. 0.9% of the global ant diversity (Bolton 2020). The list includes nine ant species endemic to Nepal and three introduced species. Twenty-one ant species were first described from Nepalese specimens. A comparison of the Nepalese ant species to those of the surrounding countries of China and India is also presented.

### Species and generic diversity within subfamily

The presented checklist is comprised of 128 named ant species belonging to 48 genera and eight subfamilies (see checklist below). The eight recorded subfamilies are Amblyoponinae, Dolichoderinae, Dorylinae, Formicinae, Leptanillinae, Myrmicinae, Ponerinae, and Pseudomyrmecinae. The ant species and genus level richness for each subfamily is given in Table 1.

**Table 1.** Number of named ant species per genus and subfamily from Nepal.

Subfamily	Genus	Number of species
Amblyoponinae	* Stigmatomma *	1
(1 genus, 1 species)		
Dolichoderinae	* Chronoxenus *	1
(4 genera, 6 species)	* Dolichoderus *	2
	* Iridomyrmex *	1
	* Technomyrmex *	2
Dorylinae	* Aenictus *	1
(3 genera, 4 species)	* Dorylus *	2
	* Ooceraea *	1
Formicinae	* Acropyga *	1
(11 genera, 36 species)	* Camponotus *	8
	* Cataglyphis *	1
	* Formica *	3
	* Lasius *	4
	* Lepisiota *	2
	* Oecophylla *	1
	* Nylanderia *	2
	* Paratrechina *	1
	* Polyrhachis *	7
	* Prenolepis *	6
Leptanillinae	* Leptanilla *	1
(1 genus, 1 species)		
Myrmicinae	* Aphaenogaster *	3
(19 genera, 63 species)	* Cardiocondyla *	7
	* Carebara *	3
	* Cataulacus *	1
	* Crematogaster *	3
	* Lophomyrmex *	1
	* Lordomyrma *	2
	* Mayriella *	1
	* Meranoplus *	3
	* Monomorium *	3
	* Myrmica *	15
	* Perissomyrmex *	1
	* Pheidole *	5
	* Pristomyrmex *	1
	* Recurvidris *	1
	* Stenamma *	1
	* Strumigenys *	8
	* Tetramorium *	3
	* Trichomyrmex *	1
Ponerinae	* Brachyponera *	2
(8 genera, 13 species)	* Diacamma *	2
	* Emeryopone *	1
	* Harpegnathos *	1
	* Leptogenys *	2
	* Odontomachus *	1
	* Odontoponera *	2
	* Pseudoneoponera *	2
Pseudomyrmecinae	* Tetraponera *	4
(1 genus, 4 species)		
**8 subfamilies**	**48 genera**	**128 species**

Myrmicinae is the most diverse subfamily in the region with 19 genera and 63 species, followed by Formicinae (11 genera and 36 species), Ponerinae (8 genera and 13 species), Dolichoderinae (4 genera and 6 species), Dorylinae (3 genera and 4 species), Pseudomyrmecinae (1 genus and 4 species), Leptanillinae (1 genus and 1 species) and Amblyoponinae (1 genus and 1 species). Over 87% of all described ant species of the world are from four main subfamilies, Myrmicinae, Formicinae, Ponerinae and Dolichoderinae (Guénard 2013). These four subfamilies are also the most speciose subfamilies of ants in Nepal, with over 92% of all described Nepalese species.

In Nepal, the most diverse ant genus is *Myrmica* with 15 valid species, followed by *Camponotus* (8), *Strumigenys* (8), *Cardiocondyla* (7), *Prenolepis* (6), *Polyrhachis* (7), *Pheidole* (5) and *Tetraponera* (4), *Lasius* (4). Eighteen other genera contain two or three species: *Formica* (3), *Nylanderia* (2) *Lepisiota* (2), *Tetramorium* (3), *Aphaenogaster* (3), *Meranoplus* (3), *Carebara* (3), *Dorylus* (2), *Brachyponera* (2), *Leptogenys* (2), *Crematogaster* (3), *Lordomyrma* (2), *Technomyrmex* (2), *Dolichoderus* (2), *Monomorium* (3), *Diacamma* (2), *Odontoponera* (2) and *Pseudoneoponera* (2). Twenty-two genera are represented in Nepal by a single species. The most speciose ant genus in India is *Camponotus* with 83 species (Bharti et al. 2016a) while that in China is *Tetramorium* with 52 species (Guénard and Dunn 2012). *Myrmica* is also a highly speciose genus in India and China with 33 and 49 species respectively but its distribution is restricted to the Himalayan region (Guénard and Dunn 2012; Bharti et al. 2016a, b). About 44% of the ant species known from Nepal are included within the seven most diverse ant genera. Similar faunistic patterns are found in the neighboring countries of India and China. The 15 most diverse genera include nearly 60% of the ant species known from China (Guénard and Dunn 2012) while seven of the most diverse genera constitute above 37% of Indian ant species (Bharti et al. 2016a).

This paper adds 84 named ant species to the list of Nepalese ants since the publication of Thapa’s 2015 “Insect diversity in Nepal” which listed 44 named ant species based upon bibliographic review. Further, this paper adds 79 named ant species to the AntWeb (2020) list and 46 named species to the AntWiki (2020) list. In light of the ant species richness found in neighboring countries (e.g., China: Guénard and Dunn 2012; India: Bharti et al. 2016a) and the ecological diversity of Nepal, many more than the 128 ant species on this checklist can be expected from Nepal. The majority of early ant collections on which the list is mainly based were done by direct hand collecting, which was not extensive. The checklist records show that the entire country is under sampled and that little sampling effort has been made in the western and southern regions in particular. To overcome this sampling bias and to establish a more thorough ant species list, we recommend that future investigations of ant diversity following standard ant sampling techniques (see Agosti and Alonso 2000) in all parts of the country. A more intensive and systematic collection of Nepalese ants would certainly add new species and new distribution records.

### New ant records for Nepal

Six ant species, namely *Harpegnathos venator*, *Monomorium pharaonis*, *Nylanderia bourbonica*, *Odontoponera denticulata*, *Polyrhachis tyrannica* and *Pseudoneoponera bispinosa* are new records for Nepal identified from the first author’s collection. The ant genus *Harpegnathos* is recorded for the first time for Nepal.

### Endemic ants of Nepal and the species described from Nepalese specimens

Of the 128 ant species recorded from Nepal, 21 species were described from Nepalese specimens and nine species are considered endemic to Nepal (Table 2). No ant genera are known to be endemic to Nepal. However, voucher specimens from previous records from Nepal were not found to be deposited in the country. Three ant species, namely *Paratrechina longicornis*, *Trichomyrmex destructor* and *Monomorium pharaonis* are globally distributed ant species that have been introduced into Nepal.

**Table 2.** Ant species first described from Nepal and species endemic to Nepal (*endemic).

*Prenolepis darlena* Williams & LaPolla, 2016	*Myrmica weberi* Elmes & Radchenko, 2009
*Prenolepis nepalensis Williams & LaPolla, 2018	*Stenamma gurkhale DuBois, 1998
*Leptanilla buddhista Baroni Urbani, 1977	*Strumigenys buddhista De Andrade, 2007
*Aphaenogaster pachei* (Forel, 1906)	*Strumigenys exilirhina* Bolton, 2000
*Mayriella transfuga* Baroni Urbani, 1977	*Strumigenys hemisobek* (Bolton, 2000)
*Meranoplus nepalensis Schödl, 1998	*Strumigenys hindu De Andrade, 2007
*Myrmica alperti* Elmes & Radchenko, 2009	*Strumigenys nepalensis* De Andrade, 1994
*Myrmica boltoni Radchenko & Elmes, 1998	*Strumigenys podarge* (Bolton, 2000)
*Myrmica brancuccii* Radchenko, Elmes & Collingwood, 1999	*Tetramorium difficile* Bolton, 1977
*Myrmica martensi Radchenko & Elmes, 1998	*Emeryopone franzi (Baroni Urbani, 1975)
*Myrmica pachei* Forel, 1906	

### Occurrence of Nepalese ant fauna in neighboring countries

A comparison of occurrence of Nepalese ants (128 species, 48 genera – this list) in the neighboring countries of China (939 species, 103 genera – Guénard and Dunn 2012) and India (828 species, 100 genera – Bharti et al. 2016a) revealed that Nepal shares 94 species with India (ca. 73% of Nepal’s fauna) and 67 species (ca. 52%) with China. Fifty-nine species are found in all three countries and 26 species are unique to Nepal. Almost half of the genera recorded from India (100) and China (103) are also recorded from Nepal (48). In terms of named and recorded species, Nepal harbors approximately 15% of the species from India and nearly 14% of the species from China. Interestingly, 47 ant species recorded from Nepal have India as the type locality while only six species have China as the type locality.

## Conclusions

An updated checklist of the ants of Nepal contains 128 valid ant species belonging to 48 genera and eight subfamilies. Six ant species (*Harpegnathos venator*, *Monomorium pharaonis*, *Nylanderia bourbonica*, *Odontoponera denticulata*, *Polyrhachis tyrannica* and *Pseudoneoponera bispinosa*) are new records for Nepal. The ant genus *Harpegnathos* is recorded for the first time for Nepal. The most speciose subfamily and genus in Nepal are Myrmicinae and *Myrmica* respectively. Overall, 21 species were first described from Nepalese specimens, nine species are endemic to Nepal, and three ant species are introduced species. No ant genera are known to be endemic to Nepal. Nepal shares over half of the listed ant species with its neighboring countries.

Despite an extensive literature review, the checklist remains incomplete because it is based on limited sampling efforts and on inadequate taxonomic knowledge of the ants of Nepal. Many regions of Nepal are clearly under sampled, such that future exploration should reveal additional species. The insufficient collections and limited published information present challenges to determining and evaluating distribution patterns of Nepalese ants (Subedi and Budha 2020). However, the checklist presented here provides a waypoint for further studies of diversity and distribution of Nepalese ants.

## Checklist

### AMBLYOPONINAE Forel, 1893

#### *Stigmatomma* Roger, 1859: 1 species

**Type-species.***Stigmatomma denticulatum* Roger, 1859: 251, **Type locality.** Island of Zante, Mount Scapo (Greece).

***Stigmatomma pertinax* (Baroni Urbani, 1978)**

**Distribution in Nepal.** Taplejung: Maewakhola (= Maiwakhola), Sanghu, KH Hyatt leg., NHMUK (AntWeb 2020).

### DOLICHODERINAE Forel, 1878

#### *Chronoxenus* Santschi, 1919: 1 species

**Type-species.***Bothriomyrmex myops* Forel, 1895: 471, **Type locality.** Kolaba, South Konkan, Maharastra, India.

***Chronoxenus dalyi* (Forel, 1895)**

**Distribution in Nepal.** Solukhumbu: Tate, 2900 m (Collingwood 1970); Nepal (Saroj et al. 2018).

#### *Dolichoderus* Lund, 1831: 2 species

**Type-species.***Formica attelaboides* Fabricius, 1775: 394, **Type locality.** Brafilia, Brazil.

***Dolichoderus affinis* Emery, 1889**

**Distribution in Nepal.** Specific locality unknown, NHMUK (Dill 2002).

***Dolichoderus taprobanae* (Smith, 1858)**

**Distribution in Nepal.** Sankhuwasabha: 16 km NE Tumlingtar, 740 m, C Carpenter leg., PSWC (AntWeb 2020).

#### *Iridomyrmex* Mayr, 1862: 1 species

**Type-species.***Formica detecta* Smith, 1858: 36, **Type locality.** Hunter river, Australia.

***Iridomyrmex anceps* (Roger, 1863)**

**Distribution in Nepal.** Gorkha, AR Sthapit leg. (Thapa 2015).

#### *Technomyrmex* Mayr, 1872: 2 species

**Type-species.***Technomyrmex strenua* Mayr, 1872: 147, **Type locality.** Sarawak, Borneo.

***Technomyrmex elatior* Forel, 1902**

**Distribution in Nepal.** Kaski: Pokhara, 760 m, PS Ward leg., PSWC (Bolton 2007; AntWeb 2020).

***Technomyrmex obscurior* Wheeler, 1928**

**Distribution in Nepal.** Kaski: 4 km SSW Pokhara, 900 m, PS Ward leg., PSWC (Bolton 2007; AntWeb 2020).

### DORYLINAE Leach, 1815

#### *Aenictus* Shuckard, 1840: 1 species

**Type-species.***Aenictus ambiguus* Shuckard, 1840: 268, **Type locality.** Punah, Bombay, India.

***Aenictus sagei* Forel, 1901**

**Distribution in Nepal.** Specific locality unknown (Jaitrong and Ruangsittichai 2018).

#### *Dorylus* Fabricius, 1793: 2 species

**Type-species.***Vespa helvola* Linnaeus, 1764: 412, **Type locality.** South Africa.

***Dorylus labiatus* Schuckard, 1840**

**Distribution in Nepal.** Lalitpur: Khumaltar, DR Sharma leg. (Thapa 2000, 2015).

***Dorylus orientalis* Westwood, 1835**

**Distribution in Nepal.** Kathmandu, N Kumar leg.; Kavre: near Banepa, 1420 m; Dolakha: Namdu, 1400 m, Sikris, 2250 m (Collingwood 1970; Thapa 2015).

#### *Ooceraea* Roger, 1862: 1 species

**Type-species.***Ooceraea fragosa* Roger, 1862: 249, **Type locality.** Sri Lanka (“Ceylon”).

***Ooceraea biroi* (Forel, 1907)**

**Distribution in Nepal.** Bara: Amlekhgunj, EI Coher leg (Wilson and Taylor 1967 as *Syscia sylvestrii*); Nepal (Ogata 1983; Wetterer et al. 2012; Bharti and Akbar 2013a).

### FORMICINAE Latreille, 1809

#### *Acropyga* Roger, 1862: 1 species

**Type-species.***Acropyga acutiventris* Roger, 1862: 243, **Type locality.** Sri Lanka.

***Acropyga yaeyamensis* Terayama & Hashimoto, 1996**

**Distribution in Nepal.** Lalitpur: Godavari (= Godawari), C Baroni Urbani leg., MCZC, NHMB (LaPolla 2004).

#### *Camponotus* Mayr, 1861: 8 species

**Type-species.***Formica ligniperda* Latreille, 1802: 88, **Type locality.** Vitrac-sur-Montane, France.

***Camponotus angusticollis* (Jerdon, 1851)**

**Distribution in Nepal.** Dolakha: Jarsa, 2000 m (Collingwood 1970; Thapa 2015).

***Camponotus compressus* (Fabricius, 1787)**

**Distribution in Nepal.** Kavrepalanchok: Chyaubas, 2000 m; Dolakha: Sikris, 2300 m, J Quinlan leg.; Kaski: Phewa lake, Pokhara, 830 m., J Quinlan leg. (Collingwood 1970; Thapa 2015); Nepal (Sheikh et al. 2017).

***Camponotus dolendus* Forel, 1892**

**Distribution in Nepal.** Dolakha: Namdu, 1450 m; Kavrepalanchok: Cha Khola, 900–1000 m, Hoxe valley; Kaski: Phewa lake, Pokhara, 830 m, J Quinlan leg. (Collingwood 1970; Thapa 2015).

***Camponotus himalayanus* Forel, 1893**

**Distribution in Nepal.** Solukhumbu: Thansindu, 3500 m (Collingwood 1970; Thapa 2015).

***Camponotus lamarckii* Forel, 1892**

**Distribution in Nepal.** Kaski: Pokhara, 1000 m, J Quinlan leg. (Collingwood 1970).

***Camponotus rufoglaucus* (Jerdon, 1851)**

**Distribution in Nepal.** Kaski: Pokhara, 1000 m, J Quinlan leg. (Collingwood 1970; Thapa 2015).

***Camponotus singularis* (Smith, 1858)**

**Distribution in Nepal.** Kaski: Phewa lake, Pokhara, 830 m, J Quinlan leg. (Collingwood 1970; Thapa 2015).

***Camponotus wroughtonii* Forel, 1893**

**Distribution in Nepal.** Ramechhap: Likhu Khola, 1690 m; Solukhumbu: Yaral, 3900 m, Pangboche, 4000 m, Thangpoche, 3500 m; Kavrepalanchok: Cha Khola, 900–1000 m, Hoxe, 1200 m (Collingwood 1970; Thapa 2015).

#### *Cataglyphis* Foerster, 1850: 1 species

**Type-species.***Cataglyphis fairmairei* Foerster, 1850: 93, **Type locality.** Algeria.

***Cataglyphis emeryi* (Karavaiev, 1911)**

**Distribution in Nepal.** Specific locality unknown (Latibari et al. 2017).

#### *Formica* Linnaeus, 1758: 3 species

**Type-species.***Formica rufa* Linnaeus, 1761: 426, **Type locality.** Sweden.

***Formica candida* Smith, 1878**

**Distribution in Nepal.** Ghyaru, 3700 m, M Granados leg., KGAC (AntWeb 2020).

***Formica fusca* Linnaeus, 1758**

**Distribution in Nepal.** Gurjakhani, 2830 m, KM Hyatt leg. (Collingwood 1970).

***Formica picea* Nylander, 1846**

**Distribution in Nepal.** Mustag (= Mustang), 4800 m (Mani and Singh 1862).

#### *Lasius* Fabricius, 1804: 4 species

**Type-species.***Formica nigra* Linnaeus, 1758: 580, **Type locality.** Europe.

***Lasius alienoflavus* Bingham, 1903**

**Distribution in Nepal.** Jumla: Talphi, H Franz leg., NHMB (Collingwood 1982).

***Lasius crinitus* (Smith, 1858)**

**Distribution in Nepal.** Solukhumbu: Junbesi, 2700 m (Collingwood 1970; Thapa 2015); Arun river valley, Duna, NHMB; Bdota, 3000 m, Lay leg., NHMB (Collingwood 1982).

***Lasius magnus* Seifert, 1992**

**Distribution in Nepal.** Induwa, Kola valley, 2000 m; Simigau, Dugong Kharka, 2100 m (Seifert 2020).

***Lasius niger* (Linnaeus, 1758)**

**Distribution in Nepal.** Myagdi: Bakhri Kharka, 1830 m, J Quinlan leg. (Collingwood 1970; Thapa 2015); Arun River Valley, Duna, 2400 m, Lay leg. (Collingwood 1982).

#### *Lepisiota* Santschi, 1926: 2 species

**Type-species.***Plagiolepis rothneyi* Forel, 1894: 415, **Type locality.** India.

***Lepisiota lunaris* (Emery, 1893)**

**Distribution in Nepal.** Dolakha: Jiri Khola valley, 1900 m; Solukhumbu: Ringmo–Junbesi, 2800 m; Ghai, 2700 m; Tate, 2900 m; Nare Ghat, 2700 m; Ramechhap, Likhu Khola, 1690 m (Collingwood 1970; Thapa 2015).

***Lepisiota watsonii* (Forel, 1894)**

**Distribution in Nepal.** Dolakha: Jiri Khola Valley, 1900 m; Solukhumbu: Ringmo–Junbesi, 2800 m (Collingwood 1970; Thapa 2015).

#### *Oecophylla* Smith, 1860: 1 species

**Type-species.***Formica virescens* Fabricius, 1775: 392, **Type locality.** Australia.

***Oecophylla smaragdina* (Fabricius, 1775)**

**Distribution in Nepal.** Kavrepalanchok/Sindhupalanchok: Charnawati/Zharange Khola to Kiratechap, 1160–1800 m, Bhotekoshi, 1150 m; Tamba Kosi, 1150–1450 m, Dhading: Dhunibesi, KC Sharma, J Hanstek and others leg. (Collingwood 1970); Gandaki zone, Turture to Phalenksangu, Marsyangdi Valley, 716 m, J Balderson leg, ANIC (AntWeb 2020); Solukhumbu: Namche bazar, 3800 m, AP Kapoor leg. (Kapoor 1961); Nepal (Wetterer 2017).

#### *Nylanderia* Emery, 1906: 2 species

**Type-species.***Formica vividula* Nylander, 1846: 900, **Type locality.** Finland.

***Nylanderia bourbonica* (Forel, 1886)**

**Distribution in Nepal.** Nagarjun forest, Shivapuri-Nagarjun National Park, 1660 m, IP Subedi leg., CDZMTU.

***Nylanderia indica* (Forel, 1894)**

**Distribution in Nepal.** Solukhumbu: Thaksindhu, 3500 m, Ringmo–Junbesi, 2800 m, Tate, 2900 m; Ramechhap: Likhu khola, 1690 m (Collingwood 1970).

#### *Paratrechina* Motschoulsky, 1863: 1 species

**Type-species.***Paratrechina currens* Motschoulsky, 1863: 14, **Type locality.** Russia.

***Paratrechina longicornis* (Latreille, 1802)**

**Distribution in Nepal.** Region del Terai: Ratnanagar, 200 m, M Granados leg., KGAC (AntWeb 2020); Bara: Amlekhganj (= Amlekhgunj), EI Coher leg., MCZ (Wetterer 2008).

#### *Polyrhachis* Smith, 1857: 6 species

**Type-species.***Formica bihamata* Fabricius, 1775: 394, **Type locality.** Insula St. Iohannis Indiae.

***Polyrhachis dives* Smith, 1857**

**Distribution in Nepal.** Arun valley, 2134 m, L Swan leg., CASC (AntWeb 2020).

***Polyrhachis illaudata* Walker, 1859**

**Distribution in Nepal.** Kaski: Phewa lake, Pokhara, 830 m, J Quinlan leg. (Collingwood 1970).

***Polyrhachis lacteipennis* Smith, 1858**

**Distribution in Nepal.** Kaski: Pokhara, 1000 m, J Quinlan leg. (Collingwood 1970); 16 km NE Baglung, 1620 m, PS Ward leg., PSWC (AntWeb 2020); Kathmandu: Basundhara, 1340 m, IP Subedi leg.; Nagarjun forest, Shivapuri-Nagarjun National Park, 1600 m, IP Subedi leg., CDZMTU.

***Polyrhachis rastellata* (Latreille, 1802)**

**Distribution in Nepal.** Nagarjun forest, Shivapuri-Nagarjun National Park, 1650 m, IP Subedi leg., CDZMTU.

***Polyrhachis thompsoni* Bingham, 1903**

**Distribution in Nepal.** Makawanpur: 14.5 km W. Hetauda, 400 m, ES Ross and DQ Cavagnaro leg., CASC (AntWeb 2020).

***Polyrhachis tibialis* Smith, 1858**

**Distribution in Nepal.** Kathmandu (Thapa 2015); Nagarjun forest, Shivapuri-Nagarjun National Park, 1400 m, IP Subedi leg., CDZMTU.

***Polyrhachis tyrannica* Smith, 1858**

**Distribution in Nepal.** Tanahun: Jamune, 530 m, IP Subedi leg., S Yamane det., CDZMTU.

#### *Prenolepis* Mayr, 1861: 6 species

**Type-species.***Tapinoma nitens* Mayr, 1853a: 144, **Type locality.** Siska, Slovenia.

***Prenolepis darlena* Williams & LaPolla, 2016**

**Distribution in Nepal.** 16 km ENE Baglung, 1100 m, PS Ward leg., USNM (Williams and LaPolla 2016).

***Prenolepis fisheri* Bharti & Wachkoo, 2012**

**Distribution in Nepal.** Kaski: 4 km SSW Pokhara, 900 m, PS Ward leg., PSWC (AntWeb 2020).

***Prenolepis fustinoda* Williams & LaPolla, 2016**

**Distribution in Nepal.** Specific locality unknown (Williams and LaPolla 2018).

***Prenolepis naoroji* Forel, 1902**

**Distribution in Nepal.** Dolakha: Jiri Khola; Kavrepalanchok: Cha Khola, Hokse valley, 1000 m; Kaski: Pokhara, 830 m, Bakhri Kharka, 1800 m, J Quinlan leg. (Collingwood 1970; Thapa 2015).

***Prenolepis nepalensis* Williams & LaPolla, 2018**

**Distribution in Nepal.** Kaski: 4 km SSW Pokhara, 900 m, PS Ward leg., USNM (Williams and LaPolla 2018).

***Prenolepis shanialena* Williams & LaPolla, 2016**

**Distribution in Nepal.** Mustang: Jomsom, 2300 m, PS Ward leg. (Williams and LaPolla 2016).

### LEPTANILLINAE Emery, 1910

#### *Leptanilla* Emery, 1870: 1 species

**Type-species.***Leptanilla revelierii* Emery, 1870: 196, **Type locality.** Corsica, France.

***Leptanilla buddhista* Baroni Urbani, 1977**

**Distribution in Nepal.** Bakhri Kharka, 1676 m, KH Hyatt leg., NHMUK; Lalitpur: Godawari (valle di Kathmandu), 1450 m, C Baroni Urbani, leg., NHMB (Baroni Urbani 1977a), Nepal (Wong and Guénard 2016).

### MYRMICINAE Lepeletier de Saint-Fargeau, 1835

#### *Aphaenogaster* Mayr, 1853: 3 species

**Type-species.***Aphaenogaster sardous* Mayr, 1853b: 107, **Type locality.** Italy.

***Aphaenogaster pachei* (Forel, 1906)**

**Distribution in Nepal.** Taplejung: Tseram, NE Nepal, 3600 m, Pache leg., MHNG (Forel 1906), Thorung Pedi, 4400 m (AntWeb 2020); Solukhumbu: Pangboche, 3950 m, Yaral and Taboche (Mingbo Valley), 3900–4800 m; Ramechhap: Likhu-Khola, 1690 m (Collingwood 1970; Thapa 2015).

***Aphaenogaster prudens* (Forel, 1902)**

**Distribution in Nepal.** Solukhumbu: Tate, 2900 m, Sikris, 2333 m, KM Hyatt leg. (Collingwood 1970; Thapa 2015).

***Aphaenogaster smythiesii* (Forel, 1902)**

**Distribution in Nepal.** Dolakha: Sikris – Jarsa, 1950–2300 m; Bakhri Kharka, 1833 m, J Quinlan leg. (Collingwood 1970; Thapa 2015).

#### *Cardiocondyla* Emery, 1869: 7 species

**Type-species.***Cardiocondyla elegans* Emery, 1869: 21, **Type locality.** Italy.

***Cardiocondyla emeryi* Forel, 1881**

**Distribution in Nepal.** Kathmandu (Seifert 2003; Wetterer 2012a).

***Cardiocondyla itsukii* Seifert, Okita & Heinze, 2017**

**Distribution in Nepal.** Kaski: Pokhara, 27 km NW (Seifert et al. 2017).

***Cardiocondyla kagutsuchi* Terayama, 1999**

**Distribution in Nepal.** Kaski: Pokhara vic., 27 km NW (Seifert 2003); Nagarjun forest, Shivapuri-Nagarjun National Park, 1400 m, IP Subedi leg., CDZMTU.

***Cardiocondyla mauritanica* Forel, 1890**

**Distribution in Nepal.** Mustang: Thak, Jomsom (Seifert 2003); Nepal (Wetterer 2012b).

***Cardiocondyla minutior* Forel, 1899**

**Distribution in Nepal.** Kathmandu, MG Allen leg., Kathmandu, Sangu (Seifert 2003; Wetterer 2014).

***Cardiocondyla obscurior* Wheeler, 1929**

**Distribution in Nepal.** Kaski: Pokhara (Seifert 2003).

***Cardiocondyla wroughtonii* (Forel, 1890)**

**Distribution in Nepal.** Specific locality unknown (Seifert 2003).

#### *Carebara* Westwood, 1840: 3 species

**Type-species.***Carebara lignata* Westwood, 1840: 86, **Type locality.** Java, Indonesia.

***Carebara bengalensis* (Forel, 1902)**

**Distribution in Nepal.** Bhaktapur (Thapa 2015).

***Carebara diversa* (Jerdon, 1851)**

**Distribution in Nepal.** Kaski: Lumle (Thapa 2015).

***Carebara lignata* Westwood, 1840**

**Distribution in Nepal.** Makawanpur: Hetauda, 330 m, W Peters leg. (Collingwood 1970).

#### *Cataulacus* Smith, 1853: 1 species

**Type-species.***Cataulacus taprobanae* Smith, 1853: 225, **Type locality.** Sri Lanka.

***Cataulacus granulatus* (Latreille, 1802)**

**Distribution in Nepal.** Baredamar, EI Coher leg (Bolton 1974).

#### *Crematogaster* Lund, 1831: 3 species

**Type-species.***Formica scutellaris* Olivier, 1792: 497, **Type locality.** France.

***Crematogaster binghamii* Forel, 1904**

**Distribution in Nepal.** Kavrepalanchok: Zharangje Khola, 1800 m (Collingwood 1970); Kathmandu, 1350 m, MG Allen leg. (Hosoishi and Ogata 2016).

***Crematogaster flava* Forel, 1886**

**Distribution in Nepal.** Specific locality unknown (Tiwari et al. 1999, 2003).

***Crematogaster himalayana* Forel, 1902**

**Distribution in Nepal.** Specific locality unknown (Tiwari et al. 1999, 2003).

#### *Lophomyrmex* Emery, 1892: 1 species

**Type-species.***Oecodoma quadrispinosa* Jerdon, 1851: 111, **Type locality.** India.

***Lophomyrmex ambiguus* Rigato, 1994**

**Distribution in Nepal.** 16 km ENE Baglung, 1100 m, PS Ward leg. (Rigato 1994; Bharti and Kumar 2012).

#### *Lordomyrma* Emery, 1897: 2 species

**Type-species.***Lordomyrma furcifera* Emery, 1897: 591, **Type locality.** New Guinea.

***Lordomyrma bhutanensis* (Baroni Urbani, 1977)**

**Distribution in Nepal.** Specific locality unknown, MCZC (Branstetter 2009).

***Lordomyrma sinensis* (Ma, Xu, Makio & DuBois, 2007)**

**Distribution in Nepal.** Specific locality unknown, MCZC (Branstetter 2009).

#### *Mayriella* Forel, 1902: 1 species

**Type-species.***Mayriella abstinens* Forel, 1902: 402, **Type locality.** Australia.

***Mayriella transfuga* Baroni Urbani, 1977**

**Distribution in Nepal.** Nawalparasi: 6 km NW Narainghat (= Narayanghat), 250 m, C Baroni Urbani leg., NHMB, NHMUK (Baroni Urbani 1977b; Shattuck and Barnett 2007; Bharti et al. 2017).

#### *Meranoplus* Smith, 1853: 3 species

**Type-species.***Cryptocerus bicolor* Guérin-Méneville, 1844: 425, **Type locality.** Pondichery, India.

***Meranoplus bicolor* (Guérin-Méneville, 1844)**

**Distribution in Nepal.** Kathmandu, 1350 m, Allen leg., NHMUK; Makawanpur: Hetaura (= Hetauda), Franz leg., NHMB; 9 miles (= 14.5 km) W Hitaura (= Hetauda), 400 m, Ross and Cavagnaro leg., CASC, Bara: Amlekhgunj, Franz leg.; Goropani, W Pokhara, NHMB (Schödl 1998); Kabre (= Kavrepalanchok), 1750 m (Collingwood 1970; Thapa 2015).

***Meranoplus nepalensis* Schödl, 1998**

**Distribution in Nepal.** Kathmandu: Gokarnaban, Gokarna Forest Reserve, 1350 m, W Wittmer and C Baroni Urbani leg., NHMB, NHMB, NHMW, MHNG, NHMUK, MCZ, Lalitpur: Godawari, 1450 m, W Wittmer and C Baroni Urbani leg., NHMB; Sankhuwasabha: Tumlingtar, 950 m, I Lobi and A Smetana leg., CASC, NHMW (Schödl 1998).

***Meranoplus rothneyi* Forel, 1902**

**Distribution in Nepal.** Sankhuwasabha: NE Kuwapani, 2250 m, Lobi and Smetana leg.; Arun Valley, 3500 m, Swan leg. (Schödl 1998).

#### *Monomorium* Mayr, 1855: 2 species

**Type-species.***Monomorium monomorium* Bolton, 1987: 287 (replacement name for *Monomorium minutum* Mayr, 1855).

***Monomorium dichroum* Forel, 1902**

**Distribution in Nepal.** Makawanpur: 9 miles (= 14.5 km) W Hitaura (= Hetauda), 400 m, ES Ross and DQ Cavagnaro leg., CASC (AntWeb 2020).

***Monomorium pharaonis* (Linnaeus, 1758)**

**Distribution in Nepal.** Tanahun: Jamune, 530 m, IP Subedi leg., S Yamane det., CDZMTU.

***Monomorium sahlbergi* Emery, 1898**

**Distribution in Nepal.** Makawanpur: 9 mi (= 14.5 km) W Hitaura (= Hetauda), 400 m, ES Ross and DQ Canvagnaro leg., CASC (AntWeb 2020).

#### *Myrmica* Latreille, 1804: 15 species

**Type-species.***Formica rubra* Linnaeus, 1758: 580, **Type locality.** Europe.

***Myrmica aimonissabaudiae* Menozzi, 1939**

**Distribution in Nepal.** Mustang: Lumleek, Ghasa-Tukhe, 16 km SW Jomsom, 2550 m (Bharti et al. 2016b).

***Myrmica alperti* Elmes & Radchenko, 2009**

**Distribution in Nepal.** Solukhumbu: Thodung, 3200 m, J Martens leg., NHMB, Jiri–Thodung, W Wittmer and C Baroni Urbani leg; Shiralaybis, Jiri ghat, 2200 m, J Martens leg., NHMB, SIZK, ELMES (Elmes and Radchenko 2009; Bharti et al. 2016b).

***Myrmica bactriana* Ruzsky, 1915**

**Distribution in Nepal.** Solukhumbu: Ringmo–Junbesi, 2800 m, Yaral (Pangboche), 3900 m, Tate, 2900 m; Sindhupalchok: Hoxe (= Hokse), 1000–2000 m (Collingwood 1970; Thapa 2015).

***Myrmica boltoni* Radchenko & Elmes, 1998**

**Distribution in Nepal.** Baglung: Dhorpatan, 3000 m, T Martens leg., Goropani, W Pokhara, H Franz leg.; 18 km NNE Baglung, 2540 m, PS Ward leg., PSWC; Sankhuwasabha: vallee d’Induwa Koa, 2000 m, Lobl and Smetana leg.; Manang: Marsyangdi, 2550 m, Mustang: Lethe, 2450–2600 m, J Martens and Ausobsky leg.; Gorkha: Chuing Khola, Meme Kharka, 3300–3400 m, J Martens and W Schwaller leg.; NHMUK, SIZK, ELMES, WARD, MARTENS, SCHULZ (Radchenko and Elmes 1998; Bharti et al. 2016b), 2 miles (= 3.2 km) SE Sikha, 2286 m, J Quinlan leg., NHMUK (AntWeb 2020).

***Myrmica brancuccii* Radchenko, Elmes & Collingwood, 1999**

**Distribution in Nepal.** Utrot, M Brancucci leg., NHMUK; Lawarai, M Brancucci leg.; Kaski: Lumle, Collingwood leg., NHMUK, NHMB, CASC, SIZK (Radchenko and Elmes 1999; Bharti et al. 2016b).

***Myrmica hecate* Weber, 1947**

**Distribution in Nepal.** Kaski: Lumle, Baira Bali von Kathmandu, Makwanpur: Daman, 2400 m (Bharti et al. 2016b).

***Myrmica indica* Weber, 1950**

**Distribution in Nepal.** Lalitpur: Phulchoki, 2600 m, W Wittmer and C Baroni Urbani leg.; Dolakha: Chordung, Jiri, 2900 m, J Martens leg.; Taplejung: Simbua Khola, vic. Lassetham, 3000–3150 m, J Martens and W Schawaller leg.; Dorhar Kharka, 2700 m, J Martens and W Schawaller leg.; Rasuwa: Thare (= Tare) Pati, Gosaikunda, H Franz leg.; Sankhuwasabha: Maghang Kharka, Makalu Barun Conservation Area, 2548 m, D Emmett and Subedi leg. Sankhuwasabha: Maghang Kharka, Makalu Barun Conservation Area, 2634 m, Alpert, Alonso and Subedi leg., NHMB, SIZK, ELMES (Elmes and Radchenko 2009; Bharti et al. 2016b); Taplejung: Maiwakhola, Sanghu, PS Ward leg., NHMUK; Sankhuwasabha: vic. Pahakhola, 2700 m, J Martens and Schwaller leg., PSAS (AntWeb 2020).

***Myrmica kozlovi* Ruzsky, 1915**

**Distribution in Nepal.** Solukhumbu: Mingbo, Mandusuwa, 4000 m (Collingwood 1970); Taplejung: Sikha, 2660 m; Sindhupalchok: Phedikhola, 1500 m; Kavrepalanchok: near Banepa, 1420 m, Resangu, 1900 m (Thapa 2015); Nepal (Radchenko and Elmes 2003).

***Myrmica martensi* Radchenko & Elmes, 1998**

**Distribution in Nepal.** Rasuwa: Gosainkunda, Sing Gyang, 3200 m, J Martens leg., NHMB (Radchenko and Elmes 1998).

***Myrmica pachei* Forel, 1906**

**Distribution in Nepal.** Taplejung: Tseram, NE Nepal, 3600 m, M Pache leg., NHMG, MCZ, Himalaya, 3600 m, MSNG, Taplejung: upper Simbu Khola valley, vic. Tseram, 3250–3350 m; Dhara und Alm Lasea, 3000–3300 m, MSNG, MHNG (Forel 1906; Bharti et al. 2016b).

***Myrmica ritae* Emery, 1889**

**Distribution in Nepal.** Kaski: Ulleri, 2000 m, J Quinlan leg. (Collingwood 1970; Thapa 2015).

***Myrmica rugosa* Mayr, 1865**

**Distribution in Nepal.** Mustang: Thakkhola, Alt-Marsa, 3100–3200 m (Bharti et al. 2016b).

***Myrmica rupestris* Forel, 1902**

**Distribution in Nepal.** Dolakha: Sikris, 2330 m, Myagdi: Gurjakhani, 2833 m, KM Hyatt leg.; Kaski: Ulleri, 2650 m, J Quinlan leg. (Collingwood 1970; Thapa 2015); Thodung via Those, 3100 m, Lalitpur: Phulchoki, 2600 m, Makawanpur: Daman, 2400 m, W Wittmer and Baroni Urbani leg.; Taplejung: Omje Kharka, NW Yamputh, 2300–2500 m, Martens and Schawaller leg., PSWC (Radchenko and Elmes 2002); Mustang: 16 km SW Jomsom, 2550 m, PS Ward leg., PSWC; Timang, 2400 m, Thanchok, 2500 m, M Granados leg., KGAC (AntWeb 2020).

***Myrmica smythiesii* Forel, 1902**

**Distribution in Nepal.** Gompa, bei Tarahot, 3400 m (Bharti et al. 2016b).

***Myrmica weberi* Elmes & Radchenko, 2009**

**Distribution in Nepal.** Sankhuwasabha: Maghang Kharka, Makalu Barun Conservation Area, 2634 m, Alpert, Alonso and Subedi leg., 2548 m, D Emmett and Subedi leg., SIZK, NHMB, ELMES; Sankhuwasabha: Chauki, 2000–3000 m, NHMB (Elmes and Radchenko 2009).

#### *Perissomyrmex* Smith, 1947: 1 species

**Type-species.***Perissomyrmex snyderi* Smith, 1947: 282, **Type locality.** Guatemala (specific locality unknown).

***Perissomyrmex monticola* Baroni Urbani & De Andrade, 1993**

**Distribution in Nepal.** Kosi, Chauki, 2600–3000 m, NHMB (Radchenko 2003), Nepal (Zhou and Huang 2006; Ogata and Okido 2007; Zheng-Hui and Cheng-Lin 2012).

#### *Pheidole* Westwood, 1839: 5 species

**Type-species.***Atta providens* Sykes, 1835: 103, **Type locality.** Orientali circa Poona, India.

***Pheidole indica* Mayr, 1879**

**Distribution in Nepal.** Tamba Koshi, 1150 m; Zarangje Khola, 1800 m; Rishengu (Collingwood 1970); Dolakha: Jiri Khola valley, 1900 m, Jiri, 1950 m, Sikris, 2000 m; Ramechhap: Likhu Khola, 1690 m; Kathmandu (Collingwood 1970; Thapa 2015).

***Pheidole jucunda* Forel, 1885**

**Distribution in Nepal.** Taplejung: Sangu, 1890 m, RL Coe leg., NHMUK (AntWeb 2020).

***Pheidole parva* Mayr, 1865**

**Distribution in Nepal.** Sankhuwasabha: 16 km NE Tumlingtar, 670 m, C Carpenter leg. (Eguchi 2008).

***Pheidole sagei* Forel, 1902**

**Distribution in Nepal.** Dolakha: Jiri, 1900 m; Solukhumbu: Taboche, 4550 m, Kavrepalanchok: Cha Khola, 900–1000 m (Collingwood 1970; Thapa 2015).

***Pheidole smythiesii* Forel, 1902**

**Distribution in Nepal.** Kaski: Pokhara, 1000 m, J Quinlan leg. (Collingwood 1970).

#### *Pristomyrmex* Mayr, 1866: 1 species

**Type-species.***Pristomyrmex pungens* Mayr, 1866: 904 (junior synonym of *Myrmica punctata* Smith, F. 1860), **Type locality.** Malakka, West Malaysia.

***Pristomyrmex sulcatus* Emery, 1895**

**Distribution in Nepal.** Likhu Khola, 1700 m; Tate, 2900 m (Collingwood 1970); Sankhuwasabha: Khandbari, Arun river, 1500–1600 m, A and Z Smetana leg. (Wang 2003).

#### *Recurvidris* Bolton, 1992: 1 species

**Type-species.***Trigonogaster recurvispinosus*Forel 1890: cix.

***Recurvidris recurvispinosa* (Forel, 1890)**

**Distribution in Nepal.** Kathmandu, MG Allen leg. (Bolton 1992; Jaitrong and Wiwatwitaya 2015).

#### *Stenamma* Westwood, 1839: 1 species

**Type-species.***Stenamma westwoodii* Westwood, 1839: 219, **Type locality.** Great Britain.

***Stenamma gurkhale* DuBois, 1998**

**Distribution in Nepal.** Lalitpur: Phulchoki, 2743 m, M Brendell leg., MCZ (Dubois 1998; Bharti et al. 2012).

#### *Strumigenys* Smith, 1860: 8 species

**Type-species.***Strumigenys mandibularis* Smith, 1860a: 72, **Type locality.** St. Paul, Brazil.

***Strumigenys buddhista* De Andrade, 2007**

**Distribution in Nepal.** Kaski: Pokhara, 820 m, Wittmer and Baroni Urbani leg., NHMB (De Andrade 2007; Baroni Urbani and De Andrade 2007).

***Strumigenys caniophanoides* De Andrade, 2007**

**Distribution in Nepal.** Sankhuwasabha: vallee d’Arun, vic. Num, 1100 m, L Lobi and A Smetana leg., MHNG (Baroni Urbani and De Andrade 2007)﻿.

***Strumigenys exilirhina* Bolton, 2000**

**Distribution in Nepal.** Taplejung: Sanghu, KH Hyatt leg., NHMUK, MCZ, MHNG, OMNH (Bolton 2000; Bharti et al. 2017; AntWeb 2020); Kathmandu, M Brendell leg., PSWC (AntWeb 2020).

***Strumigenys hemisobek* (Bolton, 2000)**

**Distribution in Nepal.** Taplejung: Maiwa Khola, Sanghu, 1981 m, KH Hyatt leg., NHMUK (Bolton 2000); Nepal (Bharti and Akbar 2013b).

***Strumigenys hindu* De Andrade, 2007**

**Distribution in Nepal.** Kaski: Pokhara, 820 m, W Wittmer and C Baroni Urbani leg., NHMB (Baroni Urbani and De Andrade 2007).

***Strumigenys membranifera* Emery, 1869**

**Distribution in Nepal.** Specific locality unknown (Wetterer 2011; Bolton 2020).

***Strumigenys nepalensis* Baroni Urbani & De Andrade, 1994**

**Distribution in Nepal.** Chitwan: 6 km NW of Narainghat (= Narayanghat), 250 m, 5 km E of Manhari, 350 m, NHMB Nepal Exped., NHMB (De Andrade 1994; Baroni Urbani and De Andrade 1994; Bharti et al. 2017).

***Strumigenys podarge* (Bolton, 2000)**

**Distribution in Nepal.** Lalitpur: Godawari, 1700 m, M Brendell, NHMUK, MCZ (AntWeb 2020).

#### *Tetramorium* Mayr, 1855: 3 species

**Type-species.***Formica caespitum* Linnaeus, 1758: 581, **Type locality.** Europe.

***Tetramorium bicarinatum* (Nylander, 1846)**

**Distribution in Nepal.** Kathmandu, MG Allen leg., NHMUK (Wetterer 2009b).

***Tetramorium difficile* Bolton, 1977**

**Distribution in Nepal.** Tamur River, Dobhan, K Hyatt leg., NHMUK, MCZ (Bolton 1977).

***Tetramorium lanuginosum* Mayr, 1870**

**Distribution in Nepal.** Bara: Amlekhgunj, EI Coher leg., MCZ (Wetterer 2010).

#### *Trichomyrmex* Mayr, 1865: 1 species

**Type-species.***Trichomyrmex rogeri* Mayr, 1865: 19, **Type locality.** Sri Lanka.

***Trichomyrmex destructor* (Jerdon, 1851)**

**Distribution in Nepal.** Taplejung, RL Coe leg., NHMUK (Wetterer 2009a); Chitwan: Ratnanagar, 200 m, M Granados leg., KGAC (AntWeb 2020).

### PONERINAE Lepeletier de Saint-Fargeau, 1835

#### *Brachyponera* Emery, 1900: 2 species

**Type-species.**Euponera (Brachyponera) luteipes croceicornis Emery, 1900: 315, **Type locality.** New Guinea.

***Brachyponera chinensis* (Emery, 1895)**

**Distribution in Nepal.** Sankhuwasabha: Makalu Barun National Park, 2400 m, G Alpert et al. leg., MCZ (Guénard et al. 2018); Nepal (Nelder et al. 2006; Ying 2013); Nagarjun forest, Shivapuri-Nagarjun National Park, 1400–1900 m, IP Subedi leg., CDZMTU.

***Brachyponera nigrita* (Emery, 1895)**

**Distribution in Nepal.** Dolakha: Jiri Khola, 1900 m; Kavrepalanchok, 1750 m (Collingwood 1970; Thapa 2015).

#### *Diacamma* Mayr, 1862: 1 species

**Type-species.***Ponera rugosa* Le Guillou, 1842: 318, **Type locality.** Borneo.

***Diacamma rugosum* (Le Guillou, 1842)**

**Distribution in Nepal.** Specific locality unknown (Tiwari et al. 2003).

***Diacamma vagans* (Smith, 1860)**

**Distribution in Nepal.** Specific locality unknown (Tiwari et al. 1999).

#### *Emeryopone* Forel, 1912: 1 species

**Type-species.***Emeryopone buttelreepeni* Forel, 1912: 318, Type species: Borneo.

***Emeryopone franzi* (Baroni Urbani, 1975)**

**Distribution in Nepal.** Kaski: Pokhara e Goropani, H Franz leg., NHMB (Baroni Urbani 1975).

#### *Harpegnathos* Jerdon, 1851: 1 species

**Type-species.***Harpegnathus saltator* Smith, 1858: 117, **Type locality.** Tellicherry, India.

***Harpegnathos venator* (Smith, 1858)**

**Distribution in Nepal.** Tanahun: Jamune, 530 m, IP Subedi leg., S Yamane det., CDZMTU.

#### *Leptogenys* Roger, 1861: 2 species

**Type-species.***Leptogenys falcigera* Roger, 1861: 42, **Type locality.** Sri Lanka.

***Leptogenys diminuta* (Smith, 1857)**

**Distribution in Nepal.** Locality unknown, 830–1900 m (Thapa 2015); Tanahun: Jamune, 530 m, IP Subedi leg., CDZMTU.

***Leptogenys sarasinorum* Forel, 1900**

**Distribution in Nepal.** Dolakha: Tamba Koshi (Namdu), 1400 m; Kaski: Phewa lake (Pokhara), 830 m, J Quinlan leg. (Collingwood 1970); Sindhupalchok: Bhotekoshi, 1900 m (Thapa 2015).

#### *Odontomachus* Latreille, 1804: 1 species

**Type-species.***Formica haematoda* Linnaeus, 1758: 582, **Type locality.** America Meridionali.

***Odontomachus monticola* Emery, 1892**

**Distribution in Nepal.** Kaski: 4 km SSW Pokhara, 900 m, PS Ward leg. (AntWeb 2020); Kathmandu: Basundhara, 1340 m; Nagarjun forest, Shivapuri-Nagarjun National Park, 1450 m, IP Subedi leg., CDZMTU.

#### *Odontoponera* Mayr, 1862: 2 species

**Type-species.***Ponera denticulata* Smith, 1858: 90, **Type locality.** Singapore.

***Odontoponera denticulata* (Smith, 1858)**

**Distribution in Nepal.** Tanahun: Jamune, 530 m, IP Subedi leg., S Yamane det., CDZMTU.

***Odontoponera transversa* (Smith, 1857)**

**Distribution in Nepal.** Kaski: Pokhara, 800 m, D Little leg. (AntWeb 2020).

#### *Pseudoneoponera* Donisthorpe, 1943: 1 species

**Type-species.***Pseudoneoponera verecundae* Donisthorpe, 1943: 439, Type species: Waigeu, Camp Nok, 2500 ft (= 762 m), New Guinea.

***Pseudoneoponera bispinosa* (Smith, 1858)**

**Distribution in Nepal.** Tanahun: Jamune, 530 m, IP Subedi leg., CDZMTU.

***Pseudoneoponera rufipes* (Jerdon, 1851)**

**Distribution in Nepal.** Dolakha: Namdu, 1450 m, Jarsa, 2000 m, Kavrepalanchok, 1750 m (Collingwood 1970; Thapa 2015); Kaski: Pokhara, 840 m, IP Subedi leg., CDZMTU.

### PSEUDOMYRMECINAE Smith, 1952

#### *Tetraponera* Smith, 1852: 4 species

**Type-species.***Tetraponera atrata* Smith, 1852: 44 (junior synonym of *Eciton nigrum* Jerdon, 1851), **Type locality.** Maharashtra, India.

***Tetraponera allaborans* (Walker, 1859)**

**Distribution in Nepal.** Dolakha: Namdu, 1450 m (Collingwood 1970), Baglung: 18 km N Baglung, 1020 m, PS Ward leg., Kathmandu: Gokarnaban, Kathmandu, W Wittmer and C Baroni Urbani leg., PSWC (AntWeb 2020); Lalitpur: Godawari, 1450 m, Bajra Barahi, Kaski: Pokhara, 820 m, W Wittmer and C Baroni Urbani leg.; Iwa Khola u. Sablako Pass, near Taplejung, 940–1200 m, Martens and Schawaller leg. (Ward 2001).

***Tetraponera binghami* (Forel, 1902)**

**Distribution in Nepal.** Bara: Amlekhgunj, 520 m, EI Coher leg., PSWC (Ward 2001, Zheng-Hui and Zheng-Qun 2004, AntWeb 2020).

***Tetraponera nigra* (Jerdon, 1851)**

**Distribution in Nepal.** Bara: Amlekhgunj, 520 m, EI Coher leg. (Ward 2001).

***Tetraponera rufonigra* (Jerdon, 1851)**

**Distribution in Nepal.** Kavrepalanchok: Cha Khola valley, 1000 m; Kaski: Pokhara, 1000 m; J Quinlan leg. (Collingwood 1970), Bagmati, Jyamira; Chitwan: Chumlingtar, JK Wetterer leg., UCDC, Janakpur, Sunkoshi river, near Khurko, L Morrison leg., PSWC, Kosi, 12 km ENE Tumlingtar, 1150 m, C Carpenter leg., Chitwan National Park, 600 m, A Hacklander leg., PSWC, Kosi, Arun valley, 610 m, L Swan leg, CASC (AntWeb 2020). Tanahun: Jamune, 530 m, IP Subedi leg., CDZMTU.

### Dubious and unverified records of ants in Nepal

Thirty-seven ant species reported in AntWiki (2020) and/or AntWeb (2020) and/or antmaps.org are not substantiated by specimens, or literature records and/or are with references unverified. These species are marked as dubious and have been excluded from the main species list. We present them below with explanation of their exclusion (Table 3).

**Table 3.** Dubious and unverified records of ants in Nepal.

Species	Reference	Explanation
** Formicinae **		
*Camponotus aethiops cachmiriensis* Emery, 1925	antmaps.org (2020)	Reference unverified
*Camponotus buddhae* Forel, 1892	antmaps.org (2020)	Reference unverified
*Camponotus siemsseni* Forel, 1901	antmaps.org (2020)	Reference unverified
*Camponotus socrates* Forel, 1904	antmaps.org (2020)	Reference unverified
*Camponotus sylvaticus basalis* Smith, 1878	antmaps.org (2020)	Reference unverified
*Camponotus sylvaticus paradichrous* Emery, 1925	AntWeb (2020)	No specimen or literature records
*Cataglyphis cugiai* Menozzi, 1939	antmaps.org (2020)	Reference unverified
*Formica sentschuensis* Ruzsky, 1915	AntWeb (2020)	No specimen or literature records
*Formica sanguinea* Latreille, 1798	antmaps.org (2020)	Reference unverified
*Formica truncorum* Fabricius, 1804	antmaps.org (2020)	Reference unverified
*Lepisiota rothneyi wroughtonii* (Forel, 1902)	AntWiki (2020)	No literature records
*Lepisiota sericea* (Forel, 1892)	antmaps.org (2020)	Reference unverified
*Plagiolepis balestrierii* Menozzi, 1939	AntWeb (2020)	No specimen or literature records
*Plagiolepis moelleri* Bingham, 1903	AntWeb (2020)	No specimen or literature records
*Plagiolepis pontii* Menozzi, 1939	AntWeb (2020)	No specimen or literature records
*Polyrhachis menelas* Forel, 1904	antmaps.org (2020)	Reference unverified
*Polyrhachis striata* Mayr, 1862	antmaps.org (2020)	Reference unverified
** Myrmicinae **		
*Aphaenogaster cristata* (Forel, 1902)	antmaps.org (2020)	Reference unverified
*Aphaenogaster sagei* (Forel, 1902)	antmaps.org (2020)	Specimen record unverified
*Aphaenogaster rothneyi* (Forel, 1902)	antmaps.org (2020)	Reference unverified
*Crematogaster afghanica* Pisarski, 1967	antmaps.org (2020)	Reference unverified
*Crematogaster buddhae* Forel, 1902	antmaps.org (2020)	Reference unverified
*Crematogaster sagei* Forel, 1903	antmaps.org (2020)	Reference unverified
*Crematogaster subdentata kaschgariensis* Forel, 1901	antmaps.org (2020)	Reference unverified
*Myrmica cachmiriensis* Forel, 1904	antmaps.org (2020)	Reference unverified
*Myrmica urbanii* Radchenko & Elmes, 1998	AntWiki (2020)	Reference unverified
*Pheidole rogersi* Forel, 1902	AntWeb (2020)	Reference unverified
*Pheidole templaria* Forel, 1902	antmaps.org (2020)	Reference unverified
*Pheidole woodmasoni* Forel, 1885	antmaps.org (2020)	Reference unverified
*Temnothorax desioi* (Menozzi, 1939)	antmaps.org (2020)	Reference unverified
*Temnothorax pamiricus* (Ruzsky, 1902)	antmaps.org (2020)	Reference unverified
*Temnothorax wroughtonii* (Forel, 1904)	antmaps.org (2020)	Reference unverified
*Trichomyrmex criniceps* (Mayr, 1879)	antmaps.org (2020)	Reference unverified
*Tetramorium elisabethae* Forel, 1904	antmaps.org (2020)	Reference unverified
*Tetramorium nursei* Bingham, 1903	antmaps.org (2020)	Reference unverified
*Tetramorium salvatum* Forel, 1902	antmaps.org (2020)	Reference unverified
** Ponerinae **		
*Diacamma rugosum sculptum* (Jerdon, 1851)	AntWiki (2020), AntWeb (2020)	No specimen or literature records
